# Enhanced Echocardiographic Assessment of the Aortic Root Structure: A Comparative Study of the Application of CT-3D Printing and Traditional Methods in the Teaching Context

**DOI:** 10.2174/011573403X391410250825054101

**Published:** 2025-08-28

**Authors:** Guobing Hu

**Affiliations:** 1 Department of Ultrasound, First Affiliated Hospital of Wannan Medical College, Wuhu 241001, Anhui, China

**Keywords:** CT-3D printing, echocardiographic assessment, aortic root structure, medical education, teaching methods, aortic valve diseases

## Abstract

**Introduction:**

Echocardiographic assessment of the aortic root structure is critical with regard to efforts to diagnose and manage aortic valve diseases. However, traditional teaching methods often fail to provide the necessary depth and practical experience for residents. This study addresses this knowledge gap by exploring the value of applying computed tomography 3-dimensional (CT-3D) printing in the context of teaching echocardiographic assessment of the aortic root structure.

**Methods:**

Between January 1, 2022, and November 30, 2024, thirty residents in the Ultrasound Department of our hospital were recruited and randomly divided into a 3D printing group and a traditional teaching group. Participants in the 3D printing group used CT-3D printed aortic root models, whereas those in the traditional teaching group relied on standard methods. The theoretical knowledge and operational skills of participants in both groups were evaluated.

**Results:**

Participants in the two groups did not differ significantly in terms of their theoretical knowledge. However, participants in the 3D printing group outperformed those in the traditional teaching group in terms of their operational skills; the 3D printing group also exhibited higher levels of teaching effectiveness satisfaction (all *p*<0.05).

**Discussion:**

Our results revealed that the use of CT-3D printed models can result in improved operational skills and increased teaching satisfaction, echoing the findings reported by other studies that have revealed enhanced learning outcomes as a result of the integration of 3D printing into medical education

**Conclusion:**

The use of CT-3D printed models significantly improved operational skill training and teaching satisfaction in the context of education in the echocardiographic assessment of the aortic root structure.

## 1 INTRODUCTION

In light of the rapid development of methods for transcatheter and transapical aortic valve replacement as well as those for traditional aortic valve replacement, preoperative, intraoperative, and postoperative assessments of the aortic root are crucial [1]. Echocardiography plays a vital role in this context, and the accuracy of such assessment is essential with respect to clinical decision-making. However, owing to the complexity of the aortic root structure, traditional teaching methods, such as slide presentations and PowerPoint (PPT) image displays, often fail to meet the needs of trainees in terms of their cognition of spatial structures [2]. These methods are less effective ways of training clinical thinking abilities and stimulating students’ interest in learning. The research on this topic thus exhibits a specific gap with regard to ways in which the intricate three-dimensional relationships associated with the aortic root can be conveyed to trainees, despite the fact that such knowledge is critical with regard to their understanding of and proficiency in echocardiography [3]. This gap highlights the need for innovative teaching methods and tools to be developed with the aim of enhancing spatial cognition and improving teaching efficiency. In recent years, the development of 3-dimensional (3D) printing technology has provided new teaching tools that can be used in the context of medical education, thus offering a potential solution to this educational challenge [4]. This technology is based on virtual or computer-generated three-dimensional models, which are processed layer by layer and manufactured by a 3D printer with the aim of creating particular objects; this approach can effectively help medical students learn about complex topics pertaining to human anatomy and tissues. The aim of this study was to address this gap by exploring the value of applying CT-3D printing in the context of teaching echocardiographic assessment of aortic root structures, specifically with the goals of enhancing the educational experience and improving the ability of trainees to understand and assess the aortic root. We hypothesize that the use of CT-3D printed models will significantly enhance the operational skills and teaching satisfaction of residents compared to traditional teaching methods in the context of echocardiographic assessment of the aortic root structure.

## 2 MATERIALS AND METHODS

### 2.1 Research Subjects

#### 2.1.1 Study Population and Design

For this study, we recruited 30 residents from the Ultrasound Department of our hospital between January 1, 2022, and November 30, 2024. These participants were randomly allocated to either a traditional teaching group or a 3D printing group; each group contained 15 residents. The average age of the residents in the CT-3D printing group was 25.5±1.2 years, and the male-to-female ratio was 7:8. The average age of the residents in the traditional teaching group was 25.3±1.3 years, and the male-to-female ratio was 7:8. In designing and conducting this study, although our primary outcomes did not show significant differences based on sex, we adhered to the SAGER (Sex and Gender Equity in Research) guidelines to ensure that sex and gender considerations were appropriately addressed throughout the research process. The study was designed in accordance with the Strengthening the Reporting of Observational Studies in Epidemiology (STROBE) guidelines with the aim of ensuring that a transparent and standardized reporting format was used in this context. Inclusion and Exclusion Criteria: Residents were included on the basis of their specialization in ultrasound and their lack of prior training in transthoracic echocardiography (TTE), which was identified as a critical way of ensuring equal baseline knowledge among participants and thus preventing bias. Residents who had previously undergone training in 3D printing were excluded to maintain consistency and control over variables that could influence the study outcomes.

#### 2.1.2 Ethical Considerations

Ethical review and approval of this study were provided by the Medical Ethics Committee of Yijishan Hospital; the ethical committee approval number was YJS20250012235. The need for informed consent was waived by the ethics committee in light of the noninvasive nature of this study as well as the absence of any direct intervention. However, all participants were provided with an information sheet that detailed the study's objectives, procedures, and potential risks and benefits, and written consent was obtained to ensure voluntary participation. By adhering to these ethical standards and obtaining the necessary approval, we aimed to maintain the highest level of integrity in our research and ensure that our participants' rights and welfare were protected.

### 2.2 Training Methods and Content

**Fig. (1):**
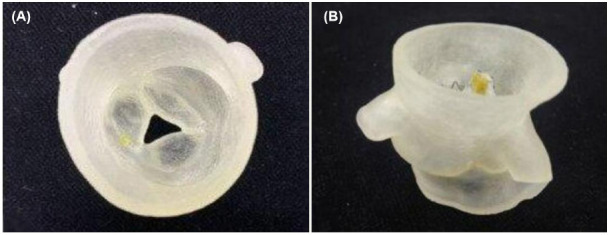
3D-printed model of the aortic root.

All the participating physicians in both groups underwent approximately 50 minutes of theoretical training in knowledge concerning the aortic root structure: experienced echocardiographers provided participants with detailed explanations of the anatomy of the aortic valve, standard cross-sections of the aortic root before transcatheter aortic valve replacement (TAVR), and measurement points. Following this theoretical training, both groups engaged in 15-minute training sessions in practical operation skills over a period of 8 weeks; these sessions were conducted twice per person per week under the expert guidance of one volunteer. The groups that participated in these sessions consisted of three people each. The 3D printing group was taught with the assistance of CT-3D printed aortic root models (Fig. **1**) during both the theoretical training and the training in practical operation skills. After these physicians completed the training, they were assessed and asked to complete satisfaction surveys that were distributed online *via* an electronic platform. These questionnaires were made available to the participants immediately following their final training session, thus ensuring that feedback was collected in a convenient and timely manner.

### 2.3 Assessment Methods and Indicators

**Fig. (2):**
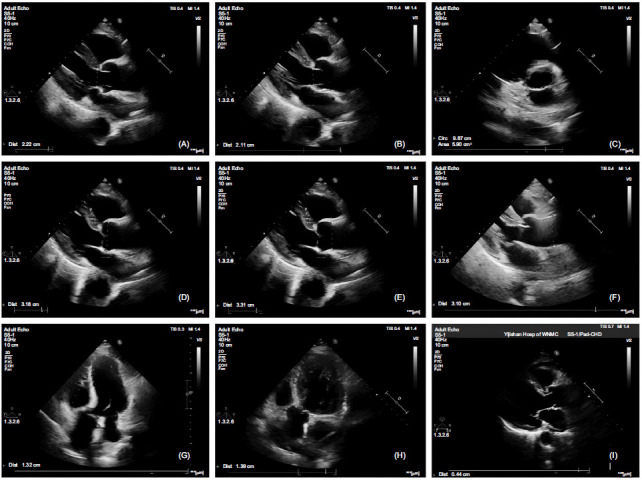
Aortic root structure measurements: **A**. left ventricular outflow tract, **B**. aortic valve ring anteroposterior diameter, **C**. aortic valve ring circumference and area, **D**. aortic sinus internal diameter, **E**. sinotubular junction internal diameter, **F**. aortic root internal diameter, **G**. left coronary artery opening height, **H**. right coronary artery opening height, **I**. anterior leaflet of the mitral valve root curtain length.

1. Assessment of Theoretical Knowledge: Immediately after the theoretical training, participants completed a test of theoretical knowledge, which consisted of multiple-choice questions, short-answer questions, and case analysis questions; a total score of 100 points was possible in each case.

2. Assessment of Operational Skills: After all the physicians completed 8 weeks of training in practical operation skills, they completed a practical operation assessment that focused on the participants’ ability to display the aortic root structure *via* TTE and obtain various measurements, including left ventricular outflow tract, aortic annulus anteroposterior diameter, aortic annulus circumference and area, aortic sinus internal diameter, sinotubular junction internal diameter, aortic root internal diameter, left coronary opening height measurement, right coronary artery opening height, and anterior leaflet of the mitral valve root curtain length (Fig. **2**). The machine used in this operation skills assessment was the Philips EPIC 7C, which is manufactured by Royal Philips, a diversified technology company that is headquartered in the Netherlands. The model used in this context was a healthy volunteer with clear images. Two ultrasound experts who had more than ten years of experience with TTE scored the training physicians; the scoring content included the quality of the cross-sectional images obtained in this context and measurement accuracy. The methods and definitions used in this assessment were based on previous studies and established guidelines for TAVR ultrasound evaluation [5]. If the image quality met the standard and the measurement results were accurate, a score of 5 points was assigned. If the image did not meet the standard or the measurement results contained errors, a score of 3 points was assigned. If assistance was needed during the process of image acquisition, a score of 1 point was assigned. If the image could not be displayed successfully, no points were awarded. Each student's final score was determined by the average score between the two judges, and total scores ranged from 0 to 45 points.

3. Satisfaction Surveys: These surveys were distributed with the goal of collecting feedback from trained doctors regarding their degree of satisfaction with the training course. The contents of the survey included adaptability to the teaching mode, teaching interactivity, acceptability, learning interest, and enthusiasm. With respect to TTE training, responses of “very satisfied” were assigned 5 points, responses of “quite satisfied” were assigned 4 points, responses of “satisfied” were assigned 3 points, responses of “neutral” were assigned 2 points, responses of “dissatisfied” were assigned 1 point, and responses of “very dissatisfied” were assigned 0 points.

### 2.4 Statistical Analysis

Data analysis was performed with the assistance of the statistical software SPSS 24.0. For normally distributed numerical data, the data are presented in terms of means ± standard deviations. Differences between groups were assessed by performing independent samples t tests with default parameters, in line with the software's standard settings. The software's default parameters were also used in this case. A *p* value less than 0.05 was considered to indicate statistical significance. We used default parameters for the SPSS 24.0 software tools mentioned in this context.

The sample size was based on an a priori power analysis conducted to achieve a desired power of 80% at a significance level of 0.05 with respect to the primary outcome measures. The sample size calculation was performed on the basis of the formula for a two-sample means test, taking into account the expected effect size on the basis of preliminary data and previous studies [6]. Specifically, the formula used in this context was as follows:

n = 
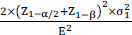
 where Z_1−α/2_ and Z_1−β_ are Z scores that correspond to the desired significance level, the power 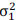 is the estimated variance, and E is the expected effect size. This calculation ensured that our study had adequate power to detect clinically significant differences between the groups.

## 3 RESULTS

A comparison of the learning effects between the two groups did not reveal any significant differences in terms of theoretical knowledge scores; however, participants in the 3D printing group significantly outperformed those in the traditional teaching group in terms of operational skills and their satisfaction with teaching effectiveness. The specific results are presented below. Importantly, no participants were excluded from the study. The study was designed to include all participants who were initially enrolled, and no participants dropped out of this research or were excluded for any reason, such as withdrawal of consent, protocol violations, or loss to follow-up.

**Fig. (3):**
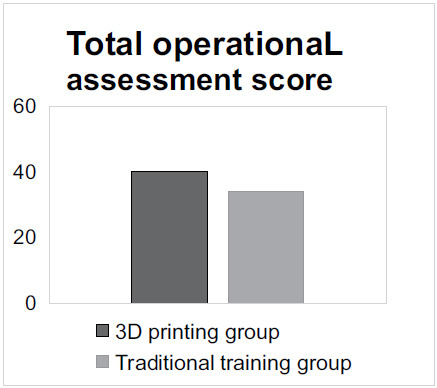
Total operational assessment scores.

**Fig. (4):**
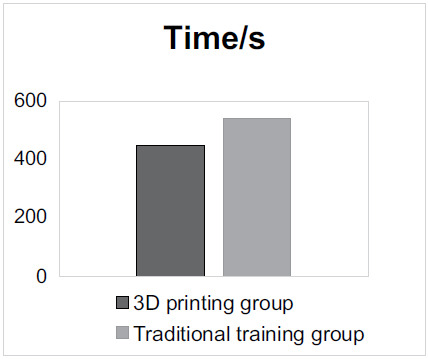
The time taken to complete all the measurements.

1. Mastery of Theoretical Knowledge: To determine whether the use of 3D printed models influenced participants’ theoretical knowledge, we compared the scores obtained by participants in both groups on the multiple-choice questions, short-answer questions, and case analysis questions, as well as their total scores. No significant differences in theoretical knowledge scores were observed between participants in the 3D printing group and those in the traditional teaching group (*p*>0.05). The scores obtained by participants in the 3D printing group were 88.73±3.39 for the multiple-choice questions, 89.93±4.23 for the short-answer questions, 85.73±4.20 for the case analysis questions, and 264.39±10.15 for the total score. The corresponding scores obtained by participants in the traditional teaching group were 86.44±3.75, 87.72±4.13, 84.42±4.18, and 258.58±9.85, respectively (Table **1**). The theoretical knowledge obtained by participants in both groups was equivalent, thus indicating that the 3D printed model did not significantly impact theoretical learning (Table **1**).

2. Operational Skills: To evaluate the impact of 3D printed models on the operational skills of the participants, we assessed their total operational assessment scores and the amount of time they took to complete all the measurements. Participants in the 3D printing group obtained significantly higher total operational assessment scores (40.50±11.50 *vs*. 34.60±8.50, *p*<0.05) and completed the measurements significantly faster (462±56 seconds *vs*. 558±68 seconds, *p*<0.05) than did participants in the traditional teaching group (Figs. **3** and **4**). Participants in the 3D printing group exhibited superior operational skills and efficiency, thus suggesting that the 3D printed model enhanced their practical learning.

Detailed Analysis of Operational Skills: To determine whether participants in the 3D printing group performed better with regard to specific measurements related to the aortic root structure, we compared the scores obtained by participants in both groups with respect to various aortic root structure measurements. Participants in the 3D printing group obtained significantly higher scores with regard to measurements related to the structures of the aortic root (*p*<0.05), which included the left ventricular outflow tract (4.7±0.8 *vs*. 4.1±0.7), aortic annulus anteroposterior diameter (4.2±0.5 *vs*. 3.6±0.6), aortic ring circumference and area (4.5±0.9 *vs*. 3.6±0.8), aortic sinus internal diameter (4.2±0.7 *vs*. 4.0±0.8), sinotubular junction internal diameter (4.4±0.8 *vs*. 4.2±0.7), aortic root internal diameter (4.6±0.7 *vs*. 4.3±0.6), left coronary artery opening (3.9±0.6 *vs*. 3.0±0.9), right coronary artery opening (4.3±0.8 *vs*. 3.4±0.8), and anterior leaflet of the mitral valve root curtain length (4.5±0.7 *vs*. 3.9±0.6; see Table **2**). Participants in the 3D printing group outperformed those in the traditional teaching group with regard to all measurements of specific operational skills, thus reinforcing the effectiveness of the 3D printed model as a way of enhancing students’ practical skills (Table **2**).

3. Comparison of Teaching Effectiveness Satisfaction between the Two Groups: To compare the satisfaction with teaching effectiveness exhibited by participants in the 3D printing group with that exhibited by participants in the traditional teaching group, we assessed adaptability to the teaching mode, teaching interactivity, acceptability, and learning interest and enthusiasm *via* satisfaction surveys. Participants in the 3D printing group reported significantly higher levels of satisfaction in all categories than did those in the traditional teaching group (*p*<0.05), as indicated by scores of 4.52±0.58 *vs*. 2.85±0.95 for adaptability, 4.44±0.63 *vs.* 2.82±0.85 for interactivity, 4.56±0.60 *vs*. 2.94±0.96 for acceptability, and 4.45±0.59 *vs*. 2.86±0.93 for learning interest and enthusiasm (Table **3**). Participants in the 3D printing group expressed greater satisfaction with teaching effectiveness, thus indicating students’ preference for the interactive and practical learning experience offered by the 3D printed model (Table **3**).

## 4 DISCUSSION

### 4.1 Enhancing the Three-dimensional Spatial Concept in Echocardiographic Teaching

Our study is in line with the extant literature on this topic, which has emphasized the limitations of traditional echocardiographic teaching methods, which frequently rely on two-dimensional slide presentations [6, 7]. These methods have been criticized in light of their inability to enable residents to obtain a robust three-dimensional spatial understanding of this context [8]. Additionally, it is important to note a significant limitation of two-dimensional TTE in the assessment of the aortic root size. TTE examination is commonly associated with systematic overestimation of the aortic diameters, particularly when measurements are performed using the “leading edge-to-leading edge” convention, compared to measurements derived from CT. This inherent limitation of TTE further underscores the need for innovative teaching tools like CT-3D printed models to enhance the accuracy and reliability of echocardiographic assessment skills among trainees.

Our finding that CT-3D printed models can significantly enhance students’ spatial comprehension of the aortic root structure contributes to our knowledge of this topic by highlighting a practical solution to this educational challenge. This advancement is consistent with previous research that has highlighted the benefits of 3D printing in medical education, particularly with regard to efforts to enhance students’ learning of complex anatomical structures [9]. Future researchers could build on our findings by exploring how the long-term retention of such a spatial understanding influences diagnostic accuracy, potentially by conducting longitudinal studies to track residents' progress over time.

### 4.2 The Roles Played by CT-3D Printing in Medical Education and Clinical Practice

Previous studies have supported our observation that CT-3D printing technology has revolutionized our ability to visualize and understand complex cardiac structures [10]. This technology has been noted for its ability to facilitate the high-fidelity replication of cardiac structures and to provide a clear display of valve abnormalities, which can be helpful in both educational and clinical settings [11]. Our study contributes to this literature by highlighting the potential of CT-3D printed models to transform two-dimensional ultrasound images into tangible forms, thereby bridging the gap between medical imaging and clinical treatment. This finding is in line with the results of previous research on the roles played by 3D printing in preoperative planning and procedural guidance [12]. Future researchers could investigate the broader integration of CT-3D printed models into clinical practice with the aim of assessing the impacts of such models on patient outcomes and procedural efficiency, as suggested by previous studies on the application of 3D printing in cardiac interventions [12].

### 4.3 Improvements in Operational Skills and Teaching Satisfaction

Our results, which revealed that the use of CT-3D printed models can result in improved operational skills and increased teaching satisfaction, echo the findings reported by other studies that have revealed enhanced learning outcomes as a result of the integration of 3D printing into medical education [13]. These findings are particularly relevant given the emphasis on practical skills in the context of echocardiography. Our study advances this field of research by providing empirical evidence to support the ability of physical models to facilitate repeated practice and encourage mastery of specific cross-sections, which represent a common challenge associated with traditional teaching methods [13]. Future researchers could extend our findings by examining the impact of this increased satisfaction and skill acquisition on students’ long-term career choices and professional development in the field of echocardiography, potentially by conducting follow-up studies with graduates who have been exposed to 3D printing during their education.

### 4.4 Limitations and Directions for Future Research

The limitations of our study, primarily with regard to its focus on entry-level TTE operation skills and the varying backgrounds in cardiology observed among participants, should be acknowledged. These limitations are consistent with a broader challenge encountered in medical education research regarding the heterogeneity of study populations [14]. Future researchers should aim to recruit a more diverse and experienced cohort to assess the broader applicability of CT-3D printed models across different levels of skill, as suggested by the need for a more comprehensive evaluation of teaching effects that has been highlighted in the literature [14]. Additionally, while our evaluation focused on ultrasound image acquisition skills, future researchers should explore the advanced interpretive skills that can be fostered by CT-3D printed models, particularly in the context of complex cardiac pathologies, as highlighted by the increasing interest in personalized medicine and precision diagnostics [14].

## CONCLUSION

**The Role of CT-3D Printed Models in Echocardiographic Education**

In conclusion, our study highlights the significant advantages offered by CT-3D printed models of the aortic root with regard to preoperative echocardiographic education. This approach not only improves teaching effectiveness and trainees’ learning experience but also has the potential to become an integral component of echocardiographic education. These findings are in line with the growing body of research that has recommended the integration of innovative technologies into medical education with the aim of enhancing learning outcomes [12]. Future researchers should explore the scalability of these findings and the potential integration of CT-3D printed models into standard echocardiographic curricula, thereby contributing to the ongoing evolution of the methodologies used in medical education.

## Figures and Tables

**Table 1:** Comparison of theoretical knowledge scores between the two groups.

	**Traditional Teaching Group**	**3D Printing Group**	**t**	***p***
Multiple-choice questions (Score)	88.73±3.39	86.44±3.75	0.988	0.33
Short-answer questions (Score)	89.93±4.23	87.72±4.13	1.448	0.16
Case analysis questions(Score)	85.73±4.20	84.42±4.18	0.856	0.40
Total Score	264.39±10.15	258.58±9.85	1.591	0.12

**Table 2:** Between-group comparison of the operation aortic root measurement skill section scores.

**Assessment section**	**3D Printing Group**	**Traditional Teaching Group**	**t**	***p***
Left ventricular outflow tract	4.7±0.8	4.1±0.7	2.186	0.037
Aortic annulus anteroposterior diameter	4.2±0.5	3.6±0.6	2.975	0.004
Aortic annulus circumference and area	4.5±0.9	3.6±0.8	2.895	0.005
Aortic sinus internal diameter	4.2±0.7	3.1±0.8	4.010	0.00111
Sinus-tube junction internal diameter	4.4±0.8	3.5±0.7	3.279	0.011
Aortic root internal diameter	4.6±0.7	3.6±0.6	4.201	0.001
Left coronary artery opening measurement	3.9±0.6	3.0±0.9	3.223	0.002
Right coronary artery opening measurement	4.3±0.8	3.4±0.8	3.081	0.004
Anterior leaflet of mitral valve root curtain measurement	4.5±0.7	3.9±0.6	2.521	0.017

**Table 3:** Comparison of teaching effectiveness satisfaction between the two groups.

**Survey Content**	**3D Printing Group**	**Traditional Teaching Group**	**t**	***p***
Adaptability to teaching mode	4.52±0.58	2.85±0.95	5.811	0.00
Teaching interactivity	4.44±0.63	2.82±0.85	5.93	0.00
Acceptability	4.56±0.60	2.94±0.96	5.54	0.00
Learning interest and enthusiasm	4.45±0.59	2.86±0.93	5.591	0.00

## Data Availability

The data and supportive information are available within the article.

## LIST OF ABBREVIATIONS

TAVR: Transcatheter Aortic Valve Replacement
STROBE: Strengthening the Reporting of Observational Studies in Epidemiology
SAGER: Sex and Gender Equity in Research
TTE: Training in Transthoracic Echocardiography

## STANDARDS OF REPORTING

STROBE guidelines were followed.
